# An updated checklist of the Tenebrionidae sec. Bousquet et al. 2018 of the Algodones Dunes of California, with comments on checklist data practices

**DOI:** 10.3897/BDJ.6.e24927

**Published:** 2018-06-14

**Authors:** M. Andrew Johnston, Rolf L Aalbu, Nico M. Franz

**Affiliations:** 1 Biodiversity Knowledge Integration Center, Arizona State University, Tempe, AZ, United States of America; 2 California Academy of Sciences, San Francisco, CA, United States of America

**Keywords:** Biodiversity informatics, checklist, Colorado Desert, darkling beetles, Darwin Core, endemism, natural history collections, occurrence data, sand dunes, Symbiota

## Abstract

Generating regional checklists for insects is frequently based on combining data sources ranging from literature and expert assertions that merely imply the existence of an occurrence to aggregated, standard-compliant data of uniquely identified specimens. The increasing diversity of data sources also means that checklist authors are faced with new responsibilities, effectively acting as filterers to select and utilize an expert-validated subset of all available data. Authors are also faced with the technical obstacle to bring more occurrences into Darwin Core-based data aggregation, even if the corresponding specimens belong to external institutions. We illustrate these issues based on a partial update of the Kimsey et al. 2017 checklist of darkling beetles - Tenebrionidae sec. Bousquet et al. 2018 - inhabiting the Algodones Dunes of California. Our update entails 54 species-level concepts for this group and region, of which 31 concepts were found to be represented in three specimen-data aggregator portals, based on our interpretations of the aggregators' data. We reassess the distributions and biogeographic affinities of these species, focusing on taxa that are precinctive (highly geographically restricted) to the Lower Colorado River Valley in the context of recent dune formation from the Colorado River. Throughout, we apply taxonomic concept labels (taxonomic name according to source) to contextualize preferred name usages, but also show that the identification data of aggregated occurrences are very rarely well-contextualized or annotated. Doing so is a pre-requisite for publishing open, dynamic checklist *versions* that finely accredit incremental expert efforts spent to improve the quality of checklists and aggregated occurrence data.

## 1. Introduction - the branching out of checklist data

Best practices of how to generate species checklists are evolving, because investments into the on-line aggregation of occurrence data (Wieczorek et al. 2012, Page et al. 2015) are generating new circumstances for creating regional biodiversity checklists (Ferro and Flick 2015, Sikes et al. 2016, GBIF 2017). For instance, at the time of preparing this article (March, 2018), the "Symbiota Collections of Arthropods Network" portal (SCAN; Gries et al. 2014, Seltmann et al. 2017) returned nearly 6.65 million occurrence records for the query "Hexapoda, USA". However, this number may only represent 5-10% of the corresponding material (estimated: >110 million) stored in North American research collections (Cobb et al. 2016). Checklist authors who strive to balance taxonomic comprehensiveness with best data science practices therefore face pragmatic choices; in effect acting as *filterers* of available data sources that range from published literature that merely imply the existence of an occurrence record, to physically vouchered but non-digitized records, to digital records that may lack a uniquely identified physical voucher and finally, to aggregated, fully standard-compliant and, hence, "research-ready" specimens (Seltmann et al. 2017). The latter often represent the most desirable minority of the available data.

Standard-formatted occurrence data are still fairly novel elements of published regional checklists, at least in the case of North American hexapod surveys. We might say that the increasing on-line presence of these data complicates the practice of creating checklists, in a good sense: they offer relevant data sources that an expert can access and potentially integrate into their checklist by querying an on-line portal. Opportunities to move such Darwin Core-compliant data from aggregator sites into peer-reviewable checklist manuscripts are becoming more widely available (e.g. Smith et al. 2013). However, doing so requires authors to apply their expertise in deciding which records and in what form, to incorporate into the checklist. Furthermore, there is also a novel social responsibility that comes with the ability to digize occurrence data. For instance, should authors be responsible for bringing on-line any non-digitized vouchered specimens from external institutions that were included in their research? In summary, the scientific and social decision tree for checklist authors is branching out in several new ways. This also means that the term *checklist* stands for an increasingly variable set of biodiversity data products, when 1-2 decades ago, it tended to refer to publications that could be fully explored off-line.

This paper aims to draw attention to some of the new scientific, technical and social aspects of checklist authorship in a Darwin Core-driven data culture. We illustrate these points based on a partial update of the Kimsey et al. 2017 checklist of insects inhabiting the Algodones Dunes of California. We limit our reassessment and discussion to the beetle family Tenebrionidae sec. (according to) Bousquet et al. 2018. Although we are critical of certain data sources and practices of Kimsey et al. (2017), our update often reflects similar pragmatic choices. It is therefore susceptible to many of the same criticisms and is far from being offered as a definitive solution to all novel checklist data representation challenges. Instead, our intention is simply to broaden the discussion of what it means to author high-quality checklists when aggregated occurrence data are available.

## 2. Taxonomic and regional background

**Note.** We follow Packer et al. (2018), who in turn cite Franz and Peet (2009), in using taxonomic concept labels - i.e. taxonomic name (author, year) *according to* source - whenever such precision is needed or desired. When only a taxonomic name is provided, this means that we accept the ambiguity that comes with this practice. For further discussion see Berendsohn (1995), Sterner and Franz (2017).

The family Tenebrionidae Latreille, 1802 sec. Bousquet et al. 2018 is a highly diverse lineage of beetles - commonly called darkling beetles - with more than 2,800 species currently recognized in North America, whose members are particularly abundant in arid habitats (Matthews et al. 2010, Thomas 1983, Bousquet et al. 2018). Their distribution includes the Algodones, or Imperial Sand Dunes, the largest active dune field in the United States located in Imperial County, California (Muhs et al. 1995, Kimsey et al. 2017). The region lies in the Lower Colorado River Valley subdivision of the Sonoran Desert, often referred to as the Colorado Desert (Shreve 1942, Shreve 1951, Brown 1994). Andrews et al. (1979) completed a landmark study of the Coleoptera sec. Bouchard et al. 2011 inhabiting sand dunes in southern California, reporting on 23 species of Tenebrionidae sec. Bousquet et al. 2018 from the Algodones. In constrast, Kimsey et al. (2017) list only four "putative endemics" of darkling beetles from these dunes.

## 3. Checklist generation methods

Faunistic studies such as Andrews et al. (1979) and Kimsey et al. (2017) have historically been generated by experts utilizing published legacy information, as well as accumulating occurrence data both from their own field work and from specimens housed in natural history collections. Frequent products of these studies have been ordered lists of taxonomic (species-level) names, which may or may not include explicit references to the underlying occurrence data (e.g. specimen label data, locally or globally unique identifiers). In addition, specimen identifications are rarely annotated with an identification source or reference to a specific taxonomic concept (Packer et al. 2018), generally the only associated information is the year of identification which, when given, may help limit the possible taxonomic concepts utilized.

Advances in biodiversity informatics are making it possible to utilize, publish and directly link taxonomic names appearing in checklists to the underlying occurrence data within a taxonomic treatment (Maddison et al. 2012, Beck et al. 2013, Smith et al. 2013). The new data sources can also introduce new uncertainties and errors, particularly regarding the consistency of taxonomic name usages (Mesibov 2013, Ferro and Flick 2015, Franz et al. 2016, Mesibov 2018). Nevertheless, occurrence-based studies should strive to make high-quality, standard-compliant biodiversity data openly available (Sikes et al. 2016).

This checklist update consists of four interconnected parts: (1) an updated novel, expert-generated list of species-level names; (2) a list of species-level names generated from aggregated occurrence data; (3) a reassessment of the apparent signals of darkling beetle endemicity in sand dunes of the arid south-western United States, including the Algodones; and (4) a critical comparison of the two checklists in the context of the expanding universe of checklist-relevant data sources.

Taxonomic and nomenclatural conventions for all checklists uniformly follow Bousquet et al. (2018). Taxonomic concept labels of the expert-generated checklist include the most congruent primary systematic reference according to which the specimens were identified.

### 3.1. Expert-generated checklist

The checklist of species-level names, published by Andrews et al. (1979), was used as the starting point for this study, with nomenclatural updates enacted to reflect the taxonomic concept labels of Bousquet et al. (2018). We then surveyed the appropriate subsequent taxonomic literature to add species-level names authoritatively reported from the Algodones; specifically: Papp (1981), Doyen (1984), Doyen (1987), MacLachlan and Olson (1990), Aalbu (2005). The checklist was completed by surveying darkling beetle specimens from the authors' personal collections, particularly the Rolf L. Aalbu Collection (henceforth: RLAC; located in California, USA), which has extensive holdings of Algodones tenebrionid material. In other words, the expert-generated checklist includes a combination of (1) literature records where no individual occurrences are explicitly recognized and (2) under-mobilized RLAC vouchers.

### 3.2. Aggregated occurrence data-based checklist

**Excluded sources.** In our assessment, the RLAC and the California State Collection of Arthropods (CSCA; located at the California Department of Food and Agriculture in Sacramento, California) are the two research collections with the most comprehensive holdings of Algodones darkling beetles. Neither of these collections currently serves occurrence data to aggregators. Meanwhile, the R.M. Bohart Museum of Entomology (UCDC; University of California, Davis), which houses the Kimsey et al. (2017) material, presently serves up data only through their institutional website: http://museums.ucdavis.edu/bohart.aspx. A total of 308 focal records were available through this website as of January 10, 2018 (Suppl. material 1). These records are not Darwin Core-compliant, however, typically lacking information on the date of collection, collector, identifier and georeference data. Therefore, they were not included in the occurrence data-based checklist. The California Terrestrial Arthropods Database (CalBug; see Hill et al. 2012; available at http://calbug.berkeley.edu/index.html) had no focal records as of January 10, 2018. Lastly, after carefully inspecting non-vouchered occurrences (observations) in select citizen science/social networks (e.g.,https://www.inaturalist.org), we were unable to confidently identify many of the photo-vouchers ourselves and judged many more non-expert identifications too doubtful to be included.

**Included sources.** Three major biodiversity data aggregators were queried for darkling beetle occurrence records from the Algodones: (1) the Symbiota Collections of Arthropod Network portal (SCAN), (2) the Integrated Digitized Biocollections portal (iDigBio) and (3) the Global Biodiversity Information Facility portal (GBIF). Records from each aggregator were downloaded on January 02, 2018. The occurrence records were sorted by the Darwin Core term "dwc:scientificName", yielding a list of unique taxonomic names and a count of the total number of records for each. All original scientific names were manually remapped to the classification of Bousquet et al. (2018). Species-level names not included in our expert-generated checklist were evaluated at the individual record level and are discussed below.

#### 3.2.1. Symbiota Collections of Arthropods Network portal

The SCAN portal (Seltmann et al. 2017; http://scan-bugs.org) was queried for "Tenebrionidae" specimen records (under taxonomic criteria and including synonyms) using the portal's Spatial Module, i.e. by specifying a geographic polygon that includes the Algodones Dunes and surrounding sandy flats. A total of 693 occurrence records were returned and then downloaded as a Darwin Core Archive (DwC-A) dataset (Suppl. material 2).

#### 3.2.2. Integrated Digitized Biocollections portal

The Integrated Digitized Biocollections portal (Page et al. 2015; https://www.idigbio.org) was queried for specimen records using the portal's map search function to draw the smallest rectangle possible covering the Algodones and using "Tenebrionidae" (search all fields), while limiting the "Basis of Record" criterion to "PreservedSpecimen". A total of 454 occurrence records were returned and then downloaded as a DwC-A dataset (Suppl. material 3). The default occurrence file (data file: occurrence.csv in the DwC-A package) was analyzed. Most of the records included a flag that the scientific name did not match the GBIF backbone taxonomy (see below), but the original data providers identifications were still returned in the scientific name field.

#### 3.2.3. Global Biodiversity Information Facility portal

The Global Biodiversity Information Facility portal (Edwards 2004; https://www.gbif.org) was queried for specimen records by adding "Tenebrionidae" as the "Scientific Name" constraint, then using the map search function under the "Location" search parameter to draw the smallest rectangle possible covering the Algodones and selecting "Preserved Specimen" under the "Basis Of Record" search criterion. A total of 133 records were returned and then downloaded as a DwC-A dataset (Suppl. material 4). The default occurrence data file delivered by GBIF only includes taxonomic names accepted in the GBIF backbone taxonomy (GBIF Secretariat 2017). No occurrence records in that default file were returned with a species-level name, but instead were matched to higher taxonomic ranks (e.g. genus level). Therefore, the verbatim records (data file: verbatim.txt in the DwC-A package) were analyzed instead of the GBIF taxonomy-validated records (data file: occurrence.txt in the DwC-A package).

## 4. Checklist results

The presentation of the checklist results follows the order of Section 3.

### 4.1. Expert-generated checklist

A total of 54 darkling beetle species-level names are included in the expert-generated Algodones checklist (Table 1). Of these, 34 were previously documented in the literature; the remaining 20 are formally published here for the first time. This increase in recognized species relative to the study of Andrews et al. (1979) (23 recorded species) is remarkable, as the new total amounts to nearly half of the 113 species-level entities reported for the entire Sonoran Desert region of California by Aalbu and Smith (2014).

Not surprisingly, access to reliable taxonomic identifications of vouchered specimens was the greatest challenge to creating the checklist, given also the scarcity of modern systematic treatments for many of the recognized species. Several groups - e.g. *Edrotes* LeConte, 1851 sec. Bousquet et al. 2018 and *Ulus* Horn, 1870 sec. Bousquet et al. 2018 - have revisions in progress, whereas others such as *Helops* Fabricius, 1775 sec. Bousquet et al. 2018 and *Hymenorus* Mulsant, 1852 sec. Bousquet et al. 2018 are in great need of revision. Hence, future studies could drastically change the species-level names and concepts employed here. Indeed, the genera *Hylocrinus* Casey, 1907 sec. Bousquet et al. 2018 and *Metoponium* Casey, 1907 sec. Bousquet et al. 2018 were last revised by Casey (1907) - a treatment that entails so many poorly differentiated species-level concepts that we know of no subsequent specialist who would confidently identify new specimens to these concepts. We similarly refrain from this task in the expert-generated checklist.

### 4.2. Aggregated occurrence data-based checklists

The results of all three aggregated occurrence data-based checklists for the Algodones darkling beetles are summarized in Table 2. The underlying raw portal data and steps taken to process and interpret them in relation to the expert-generated checklist, are provided in Suppl. material 6. Accordingly (Section I of Table 2), the SCAN portal contains 559 valid occurrences corresponding to 31 species-level concepts as recognized in Table 1 (with 108 ~ 19.3% records needing nomenclatural adjustments); the iDigBio portal serves up 386 such occurrences representing 25 species-level concepts (with 175 ~ 45.3% records needing nomenclatural adjustments; and GBIF offers 100 valid occurrences of 15 species-level concepts (with 34 ~ 34.0% needing nomenclatural adjustments).

In addition (Section II), each portal includes occurrences *not* considered valid for the focal taxonomic entities, mostly due to erroneous or uncertain identification (in our judgment), as follows: SCAN includes 133 occurrences corresponding to 21 taxonomic concepts; iDigBio contains 59 occurrences representing 21 taxonomic concepts; and GBIF serves up 34 records pertaining to 11 taxonomic concepts.

The patterns of occurrence-level overlap amongst the three data portals tell a potentially interesting story about biodiversity data meta-aggregation and signal propagation (or loss), as well as the relationship between regionally and/or taxonomically constrained portals and data quality (Mesibov 2013, Gries et al. 2014, Franz and Sterner 2018, Mesibov 2018). However, these topics reside somewhat outside of our current focus. Similarly, with the exception of the select occurrences discussed below, we will not dissect in detail the various apparent instances of nomenclatural adjustments and incorrect or uncertain identifications that the portal data represent.

#### 4.2.1. Symbiota Collections of Arthropods Network portal

Three records require in-depth discussion. First, occurrence BYUC065760 is identified in SCAN to the genus-level name *Argoporis* Horn, 1870 and located in "Vista" *County*, California, which - unlike the city of Vista (San Diego County) - is not a recognized area. Hence the georeferencing of this record is suspect. Two species of *Argoporis* sec. Berry 1980 are known from the general region (Aalbu and Smith 2014) and their members could potentially occur near the Algodones. However, the occurrence BYUC065760 is here regarded as not being a dune dweller due to the locality uncertainty and lack of other valid records.

Second and third, occurrences {X1016339, X1036349, X1012882, X1012952} are identified to the species-level name *Conibius gagates* (Horn, 1870); whereas occurrences {X1002077, X1001631} are identified to *Araeoschizus costipennis* LeConte, 1851. All six specimen identifications were made by a non-specialist and we consider them to be doubtful. There are no additional records available either via Andrews et al. 1979, Papp 1981's revision or other surveyed collections. Occurrences of *Conibius gagates* sec. Casey 1890 are otherwise known from Phoenix, Arizona and eastward thereof. We therefore cannot consider the aforementioned records as valid at this time.

See also Suppl. material 6.

#### 4.2.2. Integrated Digitized Biocollections portal

The portal propagates many of the issues originating with SCAN (see Section 4.2.1.). Occurrence BYUC087901, identified to the species-level name *Zopherus tristis* LeConte, 1851, is returned under the "Tenebrionidae" search criterion by matching an identification reference citation. However, the nominal genus has long been recognized in the family Zopheridae sec. Crowson 1955 and is classified accordingly in the iDigBio backbone taxonomy.

See also Suppl. material 6.

#### 4.2.3. Global Biodiversity Information Facility portal

The portal internally reclassifies the aggregated occurrence data specimen data according to the GBIF backbone taxonomy (GBIF Secretariat 2017). As none of the species-level names included in the expert-generated checklist is recognized in the GBIF backbone taxonomy, we could only utilize the verbatim occurrence data which pertained to only 15 species-level concepts according to our interpretation.

See also Suppl. material 6.

## 5. Precinctive tenebrionid species

Following Frank and McCoy (1990), we prefer the term *precinctive* in the sense of "confined only to the area specified", to connote a restricted geographic range, over the broader term *endemic* which can generally be applied to mean indigenous to, though the latter is often used in a synonymous sense. Two levels of precintion are assessed: (1) entities restricted to the Gran Desierto de Altar and (2) those restricted to the Lower Colorado River Valley.

Table 3 summarizes our assessment of patterns of precinction relative to the expert-generated checklist (Table 1). The patterns are based on data taken from primary literature sources; including most recently Aalbu and Smith (2014). Pertinent SCAN occurrences were added to this dataset and used to evaluate distributional boundaries. Recognized species were scored in one of three ways: (1) only known from the Algodones and the Gran Desierto de Altar; (2) only known from the Lower Colorado River Valley region of the Sonoran Desert, including at least one locality *not* within the Algodones or Gran Desierto; and (3) known to extend beyond the boundaries of the Lower Colorado River Valley. For the latter category, distributions were further differentiated as follows: (1) inhabiting the Mohave Desert; (2) inhabiting other parts of Baja California - generally the Vizcaíno region of the Sonoran Desert (see Shreve 1951, Brown 1994); and (3) inhabiting other geographic regions.

### 5.1. Gran Desierto de Altar

The nearly contiguous Algodones Dune formation and the large sand sea of the Gran Desierto de Altar are both derived from sediments from the Colorado River (Lancaster et al. 1987, Muhs et al. 1995) and are narrowly separated by the river's current course. The Colorado River begain draining into this region around 4 mya (Winker and Kidwell 1986, Derickson et al. 2008), depositing sediments that formed the Colorado River Delta, which now marks the northern limit of the Gulf of California (Waters 1983). The presently dry Salton Trough, the low-lying region north of the Colorado River Delta, has seen periodic flooding during the Holocene - by the Colorado River changing course westward and draining into the prehistoric Lake Cahuilla - at least three times in the past two thousand years (Waters 1983). Sediments from these sequential fillings of Lake Cahuilla are thought to have formed the Algodones Dunes (Norris and Norris 1961, Derickson et al. 2008). As a biogeographic factor, the Colorado River could present a barrier to gene flow and dispersal for sand-dune restricted lineages, particularly if these are flightless and thus dispersal-limited. Nevertheless, it is unclear whether any species-level entities of darkling beetles are *unique* to either the Algodones Dunes or the Gran Desierto de Altar. Moreover, historical shifts in the placement and volume of the Colorado River may have facilitated the homogenization of faunal distributions. Thus we consider the Colorado River-derived dunes - spanning both the Algodones Dunes and the Gran Desierto de Altar - as a single cohesive biogeographic region and we refer to it simply as the Gran Desierto.

As shown in Table 3, the following five entities are seemingly restricted to the Gran Desierto. *Araeoschizus andrewsi* sec. Papp 1981 and *Araeoschizus wasbauerorum* sec. Papp 1981 are both known from the Algodones and the Gran Desierto de Altar. *Batuliodes wasbaueri* sec. Doyen 1987 is known from the Algdones as well as from a small remnant sand dune area, located approximately 20 miles southeast of Mexicali, Mexico, near the Colorado River. The congruent distributions of these three flightless species reinforce the notion of a single biogeographic subregion. *Batuliomorpha imperialis* sec. Doyen 1987 and *Lepidocnemeplatia* sp. (nov.) sec. Aalbu et al. (in prep.) are both small species (~ 3 mm in length) collected mainly by sifting sand. They are currently only recorded from the Algodones, though we may expect them to be more widespread but uncollected throughout the Gran Desierto.

Kimsey et al. (2017) considered the following four species as "only recorded from the [Algodones] dunes": *Edrotes arens* sec. Doyen 1968, *Eusattus dilatatus* sec. Doyen 1984, *Nocibiotes crassipes* sec. Casey 1895 and *Tonibius sulcatus* sec. Casey 1895. We hereby refute all of these assessments of Algodones-constrained precinction. *Edrotes arens* sec. La Rivers 1947 was originally described based on three specimens from the Yuma Dunes in Arizona, with subsequent literature reports from many sand dune localities throughout California (Andrews et al. 1979). SCAN and iDigBio hold multiple occurrences of *Edrotes arens* sec. Doyen 1968 from Arizona and California localities. Specimens of *Eusattus dilatatus* sec. Doyen 1984 have been reported in literature from deep sands throughout the Lower Colorado River Valley, ranging from Puerto Peñasco, Mexico, to Blythe, California (Doyen 1984). Again, SCAN and iDigBio serve up the corresponding non-Algodones occurrences. *Nocibiotes* Casey, 1895 sec. Bousquet et al. 2018 is in need of revision, with many specimens in research collections currently not identified to the species level. However, specimens of *Nocibiotes crassipes* sec. Casey 1895 are known to occur in Baja California and throughout southern California (RLA, unpublished data). *Tonibius* Casey, 1895 sec. Bousquet et al. 2018 is presently monotypic, containing only *Tonibius sulcatus* (LeConte, 1851) sec. Casey 1895, which is the entity presumably referred to in Kimsey et al. 2017, with misattributed name authorship ("Casey"). The type locality for *Tonibius sulcatus* sec. Casey 1895 is "San Diego" (LeConte 1851) and additional occurrences are recorded in literature from Baja California (Blaisdell 1943) and Nevada (Thomas 1983). Again, SCAN and iDigBio contain respective occurrences from non-Algodones localities.

### 5.2. Lower Colorado River Valley

**Figure 1.**
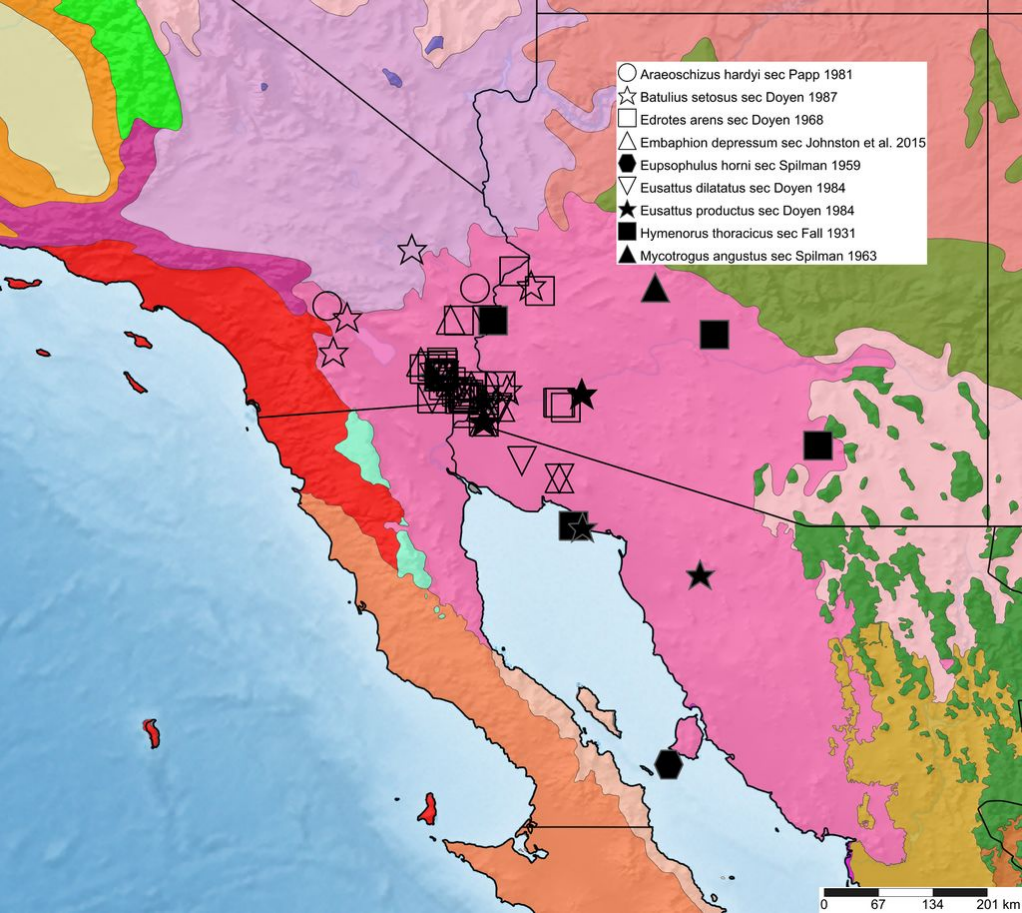
Lower Colorado River Valley Restricted Species Distributions. 239 digitized records from SCAN for 9 species. Map generated using www.simplemappr.net with background colors indicating ecoregions. The bright pink region encompassing the occurrence records roughly corresponds to the Lower Colorado River Valley subregion of the Sonoran Desert.

Nine entities present in the Algodones appear to have distributions wider than the Gran Desierto yet are still restricted to the Lower Colorado River Valley (Table 3). Two of these, *Eupsophulus horni* sec. Spilman 1959 and *Mycotrogus angustus* sec. Spilman 1963, are poorly known both in terms of their natural history and distributions. The remaining seven recognized species are typically found in areas with sandy soils. Some are only found in deeper sand dune habitats - e.g. *Edrotes arens* sec. Doyen 1968 and *Eusattus dilatatus* sec. Doyen 1984 - whereas others inhabit sandy washes and alluvial flats (e.g. *Hymenorus thoracicus* sec. Fall 1931). A total of 259 occurrences are available for these nine species in SCAN, of which 234 are considered valid in our assessment (Suppl. material 5). These occurrences are also mapped in Fig. 1 and suggest the presence of a shared distributional pattern: both towards the north, along the Colorado River and east, throughout the low desert regions of the Yuma Desert in south-western Arizona and north-western Sonora. The pattern is tentative, though plausible given similarities in habitat temperatures, rainfall and soil type. More than half of the specimens (125 occurrences) are from the well-sampled Algodones, thus offering little data regarding broader distributions of the respective species. We predict that further sampling and taxonomic identification efforts will reveal more extensive distributions for many of these.

### 5.3. Broader biogeographic relationships

The Algodones and surrounding desert environs of southern California, though usually classified as part of the Sonoran Desert (Brown 1994), have strong floristic ties to both the Mohave Desert to the north and the Vizcaíno Region in the center of the Baja California peninsula (Shreve 1942). The tenebrionid fauna of the Algodones also has strong biogeographic ties to these regions (Table 3). The strongest faunal overlap is with the Mohave Desert, which shares 29 herein recognized species with the Algodones. In contrast, only 17 species extend their distributions into non-Lower Colorado River Valley regions in Baja California. Only 28 out of the 52 examined species have ranges that extend into other biogeographic areas, which typically included either coastal California or other subregions of the Sonoran desert. This rich tenebrionid fauna of the Algodones may owe its diversity in part to the blending of psammophilic faunas from the surrounding regions.

## 6. Discussion - new opportunities for authoring checklists

### 6.1. Review of the checklist update

Regional checklists are published to be used, corrected, expanded and inevitably become outdated - the sooner the better. In that sense and only for the subcomponent of the Tenebrionidae sec. Bousquet et al. 2018, the checklist of Kimsey et al. (2017) has already served its purpose. At the same time, we have shown that these authors (and the reviewers, presumably) could have worked more thoroughly on their checklist product (see also Suppl. material 7). In addition to significant literature record omissions (e.g. Andrews et al. 1979) and nomenclatural errors, we may consider the institutional-only, non-Darwin Core database to be inadequate in the context of global biodiversity data aggregation (Maddison et al. 2012, Page et al. 2015). Moreover, occurrences of as many as 31 focal recognized species of Tenebrionidae sec. Bousquet et al. 2018 in the Algodones could have been discovered and included just by querying the SCAN portal. Indeed, every species recognized in Table 1 has at least one occurrence record in SCAN, though not necessarily from the Algodones. Thirteen species reported on SCAN from the Algodones were not listed in Andrews et al. (1979), including five which have never been reported from the region in published literature until now. In our view and considering the presence of nearly 7 million North American occurrences in SCAN currently (see Introduction), this suggests that any author, aspiring to generate a comprehensive and reliable checklist of North American insects, is well advised to explore and selectively include aggregated, occurrence data to their product. At a minimum, we would expect an explanation why such data were discarded, following their exploration (see also Ferro and Flick 2015, Sikes et al. 2016).

Of course, the flipside of the above message is this: a very considerable subsection of the Table 1 checklist depends solely on our access to and reliance on, specimen material from the Rolf L. Aalbu Collection. This collection has no on-line presence at the moment, nor foreseeable support to digitize these data moving forward. The RLAC data are both invaluable in their content and unsuited in their current form for a strictly Darwin Core-based checklist approach.

### 6.2. Evolving checklist data practices

Aggregated occurrence data typically come with a combination of data formatting and quality insufficiencies that are justly attributed to the digitizing source collection, plus other shortcomings newly generated in the process of aggregation (Mesibov 2013, Mesibov 2018, Franz and Sterner 2018). Rather than reviewing these issues (once more) in the context of our particular checklist update, we limit our discussion to a few pragmatic as well as more future-oriented solutions to enhancing occurrence data-based checklists.

We believe that the emergence of aggregated occurrence data should not only enrich the types of information sources and data formats that contribute to checklists, but should increasingly obviate altogether the notion of static, closed, print or digital checklist publications. Indeed, from a technical and perhaps also scientific point of view, the interaction between the Kimsey et al. (2017) checklist and our update need not take the form of two structurally unconnected information packages, each wholly attributed to either one or the other author team. Instead, we can envision the two respective contributions, or checklist *versions*, to develop as finely attributed bundles of annotations (Morris et al. 2013), managed on top of an underlying, unified Darwin Core-based occurrence data network. Similarities and differences between each version could then be expressed - almost entirely via automated services - as a differential ("delta" - Δ) between two Darwin Core-compatible sets of occurrence records. Subsequent authors would receive credit mainly for occurrences added, or reviewed and newly annotated, in relation to previously published records sets.

For such incremental, wholly Darwin Core-based published checklist versions to become reality, however, several aspects of authoring checklists need to receive careful attention. In particular, authors should express clearly which data sources of the current checklist version are also traceable to aggregated occurrences, or are solely reliant on expert assessment of non-mobilized records (compare Table 1 and Table 2). Our update shows that the latter category remains essential. At the same time, moving most or all occurrence records into the former category is highly desirable and a pre-requisite for fully Darwin Core standard-based checklists.

Likely, this also means that the biodiversity data community should strive to lower or remove technical and social barriers to mobilizing occurrences from private or institutional collections that currently lack the resources to accomplish aggregation. In other words, we believe that data mobilization by outsiders should become more frequent.

From a technical point of view, it is possible to set up a portal collection where any checklist author can mobilize and annotate any occurrence they are able to process as part of their research and data filtering effort - even and especially if the specimens in question belong to other individuals or institutions. We have done so, on an exploratory scale, with the "ARTSYS" collection (Externally Processed Specimens - Arthropod Systematics Research) in SCAN: http://scan-bugs.org/portal/collections/misc/collprofiles.php?collid=114. However, the prevalent culture for North American insect collections is that decisions regarding formal specimen digitization are strongly tied to the constraints of specimen ownership. This position is not well aligned with checklist author motivations to produce open, reusable data packages. An increased decoupling between the physical specimen repository and the ability to mobilize the associated occurrence data is needed.

Lastly, the notion of open, dynamic data checklists requires additional efforts to contextualize each version's - and indeed each occurrence record's - taxonomic concept usages and concept-referencing identification assertions. Too often the tradition of publishing static biodiversity data products is tied to an underlying assumption that readers will reliably understand the authors' name usages in context (though see Franz et al. 2016, Remsen 2016, Franz and Sterner 2018, Packer et al. 2018, Senderov et al. 2018).

Our use of taxonomic concept labels is one component of making checklists version-ready, by connecting the name usages in the above table to *particular* systematic treatments in which the corresponding evolutionary entities are circumscribed. Yet we should also note that, at the level of occurrences, our data are not fully there yet (see Suppl. materials 2, 3, 4). Of the 693 occurrences taken from SCAN, maximally 229 records (33.0%) entail *some* information regarding the terms dwc:identifiedBy and/or dwc:dateIdentified. Only five occurrences (0.7%) have the term dwc:identificationReference filled with data. These ratios are unsatisfactory; and yet this low degree of concept/identification reference annotation is still better in relation to the data served up by the other two aggregators. iDigBio offers 454 occurrences, which detail no identification data at all. Meanwhile GBIF has 133 records, of which 92 (69.2%) show identification data. However, these data are very frequently altered - i.e.,"elevated" to the higher-ranked taxonomic name that the GBIF taxonomy recognizes - while (falsely) retaining the original identifier attribution (see also Franz and Sterner 2018). We note in passing that only the Symbiota portal allows us to directly (via username/password log in) contribute occurrence-level identifications and taxonomic concept information.

For regional, occurrence data-based checklists to become fully open and versioning-ready, the first version should set a high bar of *decoupling* both taxonomic name usages and the identifications of occurrences from under-contextualized taxonomic names. We have attempted this for our tabular Tenebrionidae sec. Bousquet et al. 2018 of the Algodones checklist update, but are falling short regarding the underlying occurrence-level data. Moving forward, we need to treat every occurrence like a prospective micropublication that can stand on its own (see also Packer et al. 2018), by carrying sufficient taxonomic and identification-related information to be re-aggregated and re-published in updated checklist versions while retaining the provenance of its taxonomic identity and expert work effort. Only then can we assign proper credit to these experts and their work of enhancing the quality of regional checklists.

## Supplementary Material

Supplementary material 1 University of California at Davis - Bohart Museum data for the Tenebrionidae sec. Bousquet et al. 2018 from the Algodones, accessed January 10, 2018 Data type: Occurrence data in .csv format
File: oo_178920.csv M. Andrew Johnston; data provenance: Bohart Museum of Entomology, University of California, Davis

Supplementary material 2 SCAN occurrences pertinent to the checklist update Data type: Darwin Core Archive (.zip) of occurrence data and associated metadata
File: oo_178919.zip M. Andrew Johnston; data provenance: Symbiota Collections of Arthropods Network

Supplementary material 3 iDigBio occurrences pertinent to the checklist update Data type: Darwin Core Archive (.zip) of occurrence data and associated metadata
File: oo_179367.zip M. Andrew Johnston; data provenance: Integrated Digitized Biocollections

Supplementary material 4 GBIF occurrences pertinent to the checklist update Data type: Darwin Core Archive (.zip) of occurrence data and associated metadata
File: oo_178926.zip M. Andrew Johnston; data provenance: Global Biodiversity Information Facility

Supplementary material 5 SCAN occurrences for the Lower Colorado River Valley Data type: Annotated occurrence records in .csv format
Brief description: SCAN occurrences pertinent to the nine species-level entities (see Table 3) restricted to the Lower Colorado River Valley, with annotations assessing their reliability
File: oo_207371.csv M. Andrew Johnston; data provenance: Symbiota Collections of Arthropods Network

Supplementary material 6 Interpretation of species lists from biodiversity aggregators Data type: Excel spreadsheet of scientific names
Brief description: An excel workbook with three sheets. Each sheet lists the taxonomic names and record counts provided by SCAN, iDigBio and GBIF, respectively. Interpreted names according to Bousquet et al. 2018 and comments are given for each.
File: oo_190265.xlsx M. Andrew Johnston

Supplementary material 7 Interpreted list of Tenebrionidae from Kimsey et al. 2017 with comments Data type: excel spreadsheet of scientific names
Brief description: The species of tenebrionidae given in an on-line pdf checklist of the Algodones (as linked to by Kimsey et al. 2017: http://bohart.ucdavis.edu/research.html) are interpreted and commented on in light of the expert-generated checklist presented herein.
File: oo_190266.xlsx M. Andrew Johnston, Bohart Museum of Entomology, UC Davis

## Figures and Tables

**Table 1.** Expert-generated checklist of the Tenebrionidae species (sec. auctorum) known to occur in the Algodones. Records formally documented here for the first time are annotated with a "*". See Section 3 for further detail.

**Taxonomic Name (Author, Year)**	**According To (Source)**	**Information Sources**
1. *Alaephus macilentus* Casey, 1924 *	Fall 1907	RLAC
2. *Anepsius delicatulus* LeConte, 1851	Doyen 1987	Doyen 1987; RLAC
3. *Araeoschizus andrewsi* Papp, 1981	Papp 1981	Andrews et al. 1979, Papp 1981; RLAC
4. *Araeoschizus hardyi* Papp, 1981	Papp 1981	Andrews et al. 1979, Papp 1981; RLAC
5. *Araeoschizus wasbauerorum* Papp, 1981 *	Papp 1981	RLAC
6. *Asbolus laevis* LeConte, 1851	Aalbu 2005	Andrews et al. 1979, Aalbu 2005; RLAC
7. *Asbolus papillosus* (Triplehorn, 1964)	Aalbu 2005	Aalbu 2005; RLAC
8. *Asbolus verrucosus* LeConte, 1851	Aalbu 2005	Andrews et al. 1979, Aalbu 2005; RLAC
9. *Batuliodes obesus* Doyen, 1987	Doyen 1987	Doyen 1987; RLAC
10. *Batuliodes wasbaueri* Doyen, 1987	Doyen 1987	Doyen 1987; RLAC
11. *Batuliomorpha imperialis* Doyen, 1987	Doyen 1987	Doyen 1987; RLAC
12. *Batulius setosus* LeConte, 1851	Doyen 1987	Doyen 1987; RLAC
13. *Blapstinus histricus* Casey, 1890	Davis 1970	Davis 1970; RLAC
14. *Cerenopus concolor* LeConte, 1851	Berry 1973	Andrews et al. 1979; RLAC
15. *Cheirodes californicus* (Horn, 1870)	Horn 1870	Andrews et al. 1979; RLAC
16. *Chilometopon abnorme* (Horn,1870)	MacLachlan and Olson 1990	MacLachlan and Olson 1990; RLAC
17. *Chilometopon brachystomum* Doyen, 1983	MacLachlan and Olson 1990	MacLachlan and Olson 1990; RLAC
18. *Chilometopon helopioides* Horn, 1974	MacLachlan and Olson 1990	MacLachlan and Olson 1990; RLAC
19. *Chilometopon pallidum* Casey, 1890	MacLachlan and Olson 1990	MacLachlan and Olson 1990; RLAC
20. *Cnemodinus testaceus* (Horn, 1870)	Casey 1907	Andrews et al. 1979; RLAC
21. *Conibiosoma elongatum* (Horn, 1870) *	Casey 1890	RLAC
22. *Conibius opacus* (LeConte, 1866) *	Casey 1890	RLAC
23. *Craniotus pubescens* LeConte, 1851 *	Aalbu et al. 2015	RLAC
24. *Cryptoglossa muricata* (LeConte, 1851)	Aalbu 2005	Aalbu 2005; RLAC
25. *Edrotes arens* La Rivers, 1947	Doyen 1968	Andrews et al. 1979; RLAC
26. *Edrotes ventricosus* LeConte, 1851	Doyen 1968	Andrews et al. 1979; RLAC
27. *Eleodes armata* LeConte, 1851	Johnston et al. 2015	Andrews et al. 1979; RLAC
28. *Embaphion depressum* (LeConte, 1851)	Johnston et al. 2015	Andrews et al. 1979; RLAC
29. *Eupsophulus castaneus* (Horn, 1870)	Spilman 1959	Andrews et al. 1979; RLAC
30. *Eupsophulus horni* (Champion, 1885) *	Spilman 1959	RLAC
31. *Eusattus dilatatus* LeConte, 1851	Doyen 1984	Andrews et al. 1979, Doyen 1984; RLAC
32. *Eusattus productus* LeConte, 1858	Doyen 1984	Doyen 1984; RLAC
33. *Helops arizonensis* Horn, 1874 *	Horn 1874	RLAC
34. *Hylocrinus* sp. *	Casey 1907	RLAC
35. *Hymenorus exiguus* Casey, 1891 *	Fall 1931	RLAC
36. *Hymenorus irritus* Fall, 1931 *	Fall 1931	RLAC
37. *Hymenorus thoracicus* Fall, 1931 *	Fall 1931	RLAC
38. *Latheticus prosopis* Chittenden, 1904	Chittenden 1904	Andrews et al. 1979; RLAC
39. *Lepidocnemeplatia* sp. (nov.) *	Aalbu et al. (in prep.)	RLAC
40. *Lepidocnemeplatia sericia* (Horn, 1870)	Aalbu et al. (in prep.)	Andrews et al. 1979; RLAC
41. *Mecysmus angustus* (LeConte, 1851)	Thomas 1890	Andrews et al. 1979; RLAC
42. *Metoponium* sp. *	Thomas 1907	RLAC
43. *Mycotrogus angustus* Horn, 1870 *	Spilman 1963	RLAC
44. *Nocibiotes crassipes* (Casey, 1890) *	Casey 1895	RLAC
45. *Nocibiotes granulatus* (LeConte, 1851)	Thomas 1895	Andrews et al. 1979; RLAC
46. *Notibius puberulus* LeConte, 1851	Horn 1894	Andrews et al. 1979; RLAC
47. *Stenomorpha confluens* (LeConte, 1851)	Triplehorn and Brown 1971	Andrews et al. 1979; RLAC
48. *Stenomorpha hirsuta* (LeConte, 1851)	Casey 1912	Andrews et al. 1979; RLAC
49. *Telabis serrata* (LeConte, 1866) *	Casey 1890	RLAC
50. *Tonibius sulcatus* (LeConte, 1851) *	Casey 1895	RLAC
51. *Tribolium castaneum* (Herbst, 1797) *	Hinton 1948	RLAC
52. *Trichoton sordidum* (LeConte, 1851) *	Casey 1890	RLAC
53. *Triorophus laevis* LeConte, 1851 *	Horn 1870	RLAC
54. *Ulus crassus* (LeConte, 1851)	Casey 1890	Andrews et al. 1979; RLAC

**Table 2.** Summary of the aggregated occurrence (specimen) data for Algodones Tenebrionidae species (sec. auctorum) available through the SCAN, iDigBio and GBIF portals, respectively. Totals include occurrences identified to synonymous or misspelled names in relation to herein accepted source. The table is arranged in two sections for occurrences considered valid and invalid, respectively and for various reasons in the latter case. "syn." = synonym; "lap." = lapsus. See also Table 1 and Section 3.

**Taxonomic concept label**	**SCAN**	**iDigBio**	**GBIF**
**I. Occurrences considered *valid*** (including identifications to synonymous or misspelled names)			
1. *Alaephus macilentus* Casey, 1924 sec. Fall 1907	–	–	–
2. *Anepsius delicatulus* LeConte, 1851 sec. Doyen 1987	3	3	3
3. *Araeoschizus andrewsi* Papp, 1981 sec. Papp 1981	37	22	1
4. *Araeoschizus hardyi* Papp, 1981 sec. Papp 1981	3	3	–
5. *Araeoschizus wasbauerorum* Papp, 1981 sec. Papp 1981	1	1	–
6. *Asbolus laevis* LeConte, 1851 sec. Aalbu 2005	133 (25 syn.)	44 (9 syn.)	5 (5 syn.)
7. *Asbolus papillosus* (Triplehorn, 1964) sec. Aalbu 2005	7 (1 syn.)	–	–
8. *Asbolus verrucosus* LeConte, 1851 sec. Aalbu 2005	43 (8 syn.)	13	6
9. *Batuliodes obesus* Doyen, 1987 sec. Doyen 1987	–	–	–
10. *Batuliodes wasbaueri* Doyen, 1987 sec. Doyen 1987	–	–	–
11. *Batuliomorpha imperialis* Doyen, 1987 sec. Doyen 1987	6	6	–
12. *Batulius setosus* LeConte, 1851 sec. Doyen 1987	2	–	1
13. *Blapstinus histricus* Casey, 1890 sec. Davis 1970	2	1	–
14. *Cerenopus concolor* LeConte, 1851 sec. Berry 1973	10	10	9
15. *Cheirodes californicus* (Horn, 1870) sec. Horn 1870	–	–	
16. *Chilometopon abnorme* (Horn, 1870) sec. MacLachlan and Olson 1990	7	6	–
17. *Chilometopon brachystomum* Doyen, 1983 sec. MacLachlan and Olson 1990	–	–	–
18. *Chilometopon helopioides* Horn, 1974 sec. MacLachlan and Olson 1990	–	–	–
19. *Chilometopon pallidum* Casey, 1890 sec. MacLachlan and Olson 1990	19	16	–
20. *Cnemodinus testaceus* (Horn, 1870) sec. Casey 1907	43	1	–
21. *Conibiosoma elongatum* (Horn, 1870) sec. Casey 1890	–	–	–
22. *Conibius opacus* (LeConte, 1866) sec. Casey 1890	–	–	–
23. *Craniotus pubescens* LeConte, 1851 sec. Aalbu et al. 2015	–	–	–
24. *Cryptoglossa muricata* (LeConte, 1851) sec. Aalbu 2005	18 (16 syn.)	17 (16 syn.)	15
25. *Edrotes arens* La Rivers, 1947 sec. Doyen 1968	55 (2 lap.)	23 (2 lap.)	6 (2 lap.)
26. *Edrotes ventricosus* LeConte, 1851 sec. Doyen 1968	51	23	9
27. *Eleodes armata* LeConte, 1851 sec. Johnston et al. 2015	44 (39 lap.)	142 (137 lap.)	28 (24 syn.)
28. *Embaphion depressum* (LeConte, 1851) sec. Johnston et al. 2015	8	11	4
29. *Eupsophulus castaneus* (Horn, 1870) sec. Spilman 1959	16 (1 lap.)	14 (1 lap.)	1
30. *Eupsophulus horni* (Champion, 1885) sec. Spilman 1959	–	–	–
31. *Eusattus dilatatus* LeConte, 1851 sec. Doyen 1984	22 (3 syn.)	11 (3 syn.)	3
32. *Eusattus productus* LeConte, 1858 sec. Doyen 1984	1	–	–
33. *Helops arizonensis* Horn, 1874 sec. Horn 1874	–	–	–
34. *Hylocrinus* sp. sec. Casey 1907	–	–	–
35. *Hymenorus exiguus* Casey, 1891 sec. Fall 1931	–	–	–
36. *Hymenorus irritus* Fall, 1931 sec. Fall 1931	–	–	–
37. *Hymenorus thoracicus* Fall, 1931 sec. Fall 1931	–	–	–
38. *Latheticus prosopis* Chittenden, 1904 sec. Chittenden 1904	–	–	–
39. *Lepidocnemeplatia* sp. (nov.) sec. Aalbu et al. (in prep.)	3 (3 syn.)	3 (3 syn.)	3 (3 syn.)
40. *Lepidocnemeplatia sericia* (Horn, 1870) sec. Aalbu et al. (in prep.)	7	–	–
41. *Mecysmus angustus* (LeConte, 1851) sec. Casey 1890	1	–	–
42. *Metoponium* sp. sec. Casey 1907	–	–	–
43. *Mycotrogus angustus* Horn, 1870 sec. Spilman 1963	–	–	–
44. *Nocibiotes crassipes* (Casey, 1890) sec. Casey 1895	–	–	–
45. *Nocibiotes granulatus* (LeConte, 1851) sec. Casey 1895	–	–	–
46. *Notibius puberulus* LeConte, 1851 sec. Horn 1894	6 (4 syn.)	8 (4 syn.)	–
47. *Stenomorpha confluens* (LeConte, 1851) sec. Triplehorn and Brown 1971	15 (6 syn.)	6	6
48. *Stenomorpha hirsuta* (LeConte, 1851) sec. Casey 1912	2	1	–
49. *Telabis serrata* (LeConte, 1866) sec. Casey 1890	3	1	–
50. *Tonibius sulcatus* (LeConte, 1851) sec. Casey 1895	–	–	–
51. *Tribolium castaneum* (Herbst, 1797) sec. Hinton 1948	–	–	–
52. *Trichoton sordidum* (LeConte, 1851) sec. Casey 1890	–	–	–
53. *Triorophus laevis* LeConte, 1851 sec. Horn 1870	1	1	–
54. *Ulus crassus* (LeConte, 1851) sec. Casey 1890	–	–	–
**Totals**	**569** (108 syn./lap.)	**386** (175 syn./lap.)	**100** (34 syn./lap.)
**II. Occurrences considered *invalid*** (including misidentifications, misspellings and uncertain identifications)			
1. [*Araeoschizus costipennis* sec. Bousquet et al. 2018] – misidentified	2	2	–
2. [*Conibius gagates* sec. Bousquet et al. 2018] – misidentified	4	4	–
3. [*Leptohoplia* sp.] – *not* a darkling beetle	5	5	–
4. [*Argoporis* sp. sec. Bousquet et al. 2018] – not a sand dune dweller	1	1	–
5. [*Chilometopon* sp. sec. Bousquet et al. 2018] – misspelled name	2	2	–
6. [*Telabis* sp. sec. Bousquet et al. 2018] – misspelled name	4	4	–
7. [Anepsiini sp. sec. Bousquet et al. 2018] – uncertain identification	4	–	–
8. [*Cheirodes* sp. sec. Bousquet et al. 2018] – uncertain identification	1	1	1
9. [*Batuliodes* sp. sec. Bousquet et al. 2018] – uncertain identification	3	3	3
10. [*Batulius* sp. sec. Bousquet et al. 2018] – uncertain identification	3	3	3
11. [*Chilometopon* sp. sec. Bousquet et al. 2018] – uncertain identification	2	1	2
12. [*Cnemodinus* sp. sec. Bousquet et al. 2018] – uncertain identification	3	3	3
13. [*Cryptoglossa* sp. sec. Bousquet et al. 2018] – uncertain identification	1	1	–
14. [*Edrotes* sp. sec. Bousquet et al. 2018] – uncertain identification	30	6	4
15. [*Eleodes* sp. sec. Bousquet et al. 2018] – uncertain identification	1	1	1
16. [*Eusattus* sp. sec. Bousquet et al. 2018] – uncertain identification	1	1	–
17. [*Notibius* sp. sec. Bousquet et al. 2018] – uncertain identification	1	1	–
18. [Pimeliinae sp. sec. Bousquet et al. 2018] – uncertain identification	2	2	2
19. [*Telabis* sp. sec. Bousquet et al. 2018] – uncertain identification	5	5	3
20. [Tenebrionidae sp. sec. Bousquet et al. 2018] – uncertain identification	58	11	11
21. [*Triorophus* sp.sec. Bousquet et al. 2018] – uncertain identification	1	1	1
22. [*Zopherus tristis* LeConte, 1851] – *not* a darkling beetle	–	1	–
**Totals**	**133**	**59**	**34**

**Table 3.** Pattern of precinction of Tenebrionidae species (sec. auctorum) known to occur in the Algodones. Taxonomic concept labels are numbered in accordance with Tables 1, 2 to facilitate comparisons. Abbreviations: Gran Desierto = Gran Desierto de Altar; Lower Col. RV = Lower Colorado River Valley; Baja Calif. = Baja California. See text for further detail.

**Taxonomic concept label**	**Gran** **Desierto**	**Lower Col. RV**	**Mohave** **Desert**	**Baja** **Calif.**	**Other** **Areas**
3. *Araeoschizus andrewsi* Papp, 1981 sec. Papp 1981	+				
5. *Araeoschizus wasbauerorum* Papp, 1981 sec. Papp 1981	+				
10. *Batuliodes wasbaueri* Doyen, 1987 sec. Doyen 1987	+				
11. *Batuliomorpha imperialis* Doyen, 1987 sec. Doyen 1987	+				
39. *Lepidocnemeplatia* sp. (nov.) sec. Aalbu et al. (in prep.)	+				
4. *Araeoschizus hardyi* Papp, 1981 sec. Papp 1981		+			
12. *Batulius setosus* LeConte, 1851 sec. Doyen 1987		+			
25. *Edrotes arens* La Rivers, 1947 sec. Doyen 1968		+			
28. *Embaphion depressum* (LeConte, 1851) sec. Johnston et al. 2015		+			
30. *Eupsophulus horni* (Champion, 1885) sec. Spilman 1959		+ (?)			
31. *Eusattus dilatatus* LeConte, 1851 sec. Doyen 1984		+			
32. *Eusattus productus* LeConte, 1858 sec. Doyen 1984		+			
37. *Hymenorus thoracicus* Fall, 1931 sec. Fall 1931		+			
43. *Mycotrogus angustus* Horn, 1870 sec. Spilman 1963		+ (?)			
1. *Alaephus macilentus* Casey, 1924 sec. Fall 1907			+	+	+
2. *Anepsius delicatulus* LeConte, 1851 sec. Doyen 1987			+	+	+
6. *Asbolus laevis* LeConte, 1851 sec. Aalbu 2005			+		
7. *Asbolus papillosus* (Triplehorn, 1964) sec. Aalbu 2005			+		
8. *Asbolus verrucosus* LeConte, 1851 sec. Aalbu 2005			+	+	+
9. *Batuliodes obesus* Doyen, 1987 sec. Doyen 1987			+		
13. *Blapstinus histricus* Casey, 1890 sec. Davis 1970			+		+
14. *Cerenopus concolor* LeConte, 1851 sec. Berry 1973			+	+	
15. *Cheirodes californicus* (Horn, 1870) sec. Horn 1870			+		+
16. *Chilometopon abnorme* (Horn, 1870) sec. MacLachlan and Olson 1990			+	+	+
17. *Chilometopon brachystomum* Doyen, 1983 sec. MacLachlan and Olson 1990			+	+	+
18. *Chilometopon helopioides* Horn, 1974 sec. MacLachlan and Olson 1990			+	+	+
19. *Chilometopon pallidum* Casey, 1890 sec. MacLachlan and Olson 1990			+	+	+
20. *Cnemodinus testaceus* (Horn, 1870) sec. Casey 1907			+		
21. *Conibiosoma elongatum* (Horn, 1870) sec. Casey 1890			+		+
22. *Conibius opacus* (LeConte, 1866) sec. Casey 1890				+	
23. *Craniotus pubescens* LeConte, 1851 sec. Aalbu et al. 2015			+	+	+
24. *Cryptoglossa muricata* (LeConte, 1851) sec. Aalbu 2005			+	+	
26. *Edrotes ventricosus* LeConte, 1851 sec. Doyen 1968			+		+
27. *Eleodes armata* LeConte, 1851 sec. Johnston et al. 2015			+	+	+
29. *Eupsophulus castaneus* (Horn, 1870) sec. Spilman 1959			+		+
33. *Helops arizonensis* Horn, 1874 sec. Horn 1874					+
35. *Hymenorus exiguus* Casey, 1891 sec. Fall 1931					+
36. *Hymenorus irritus* Fall, 1931 sec. Fall 1931					+
38. *Latheticus prosopis* Chittenden, 1904 sec. Chittenden 1904					+
40. *Lepidocnemeplatia sericia* (Horn, 1870) sec. Aalbu et al. (in prep.)			+		+
41. *Mecysmus angustus* (LeConte, 1851) sec. Casey 1890					+
44. *Nocibiotes crassipes* (Casey, 1890) sec. Casey 1895				+	
45. *Nocibiotes granulatus* (LeConte, 1851) sec. Casey 1895					+
46. *Notibius puberulus* LeConte, 1851 sec. Horn 1894			+		+
47. *Stenomorpha confluens* (LeConte, 1851) sec. Triplehorn and Brown 1971			+		
48. *Stenomorpha hirsuta* (LeConte, 1851) sec. Casey 1912					+
49. *Telabis serrata* (LeConte, 1866) sec. Casey 1890			+	+	+
50. *Tonibius sulcatus* (LeConte, 1851) sec. Casey 1895			+	+	
51. *Tribolium castaneum* (Herbst, 1797) sec. Hinton 1948			+	+	+
52. *Trichoton sordidum* (LeConte, 1851) sec. Casey 1890			+		+
53. *Triorophus laevis* LeConte, 1851 sec. Horn 1870			+		+
54. *Ulus crassus* (LeConte, 1851) sec. Casey 1890			+	+	+
**Totals**	**5**	**9 (2?)**	**29**	**17**	**28**
